# National and provincial burden of varicella disease and cost-effectiveness of childhood varicella vaccination in China from 2019 to 2049: a modelling analysis

**DOI:** 10.1016/j.lanwpc.2022.100639

**Published:** 2022-11-11

**Authors:** Huangyufei Feng, Haijun Zhang, Chao Ma, Haonan Zhang, Dapeng Yin, Hai Fang

**Affiliations:** a School of Public Health, Peking University, Beijing, 100191, China; b China Center for Health Development Studies, Peking University, Beijing, 100191, China; c National Immunization Program, Chinese Center for Disease Control and Prevention, Beijing, 102206, China; d Hainan Center for Disease Control and Prevention, Hainan, 570203, China; e Peking University Health Science Center, Chinese Center for Disease Control and Prevention Joint Center for Vaccine Economics, Beijing, 100191, China; f Institute for Global Health and Development, Peking University, Beijing, 100191, China

**Keywords:** Varicella vaccine, Disease burden, Cost-effectiveness, China

## Abstract

**Background:**

In China, varicella is the third most frequently reported vaccine-preventable infectious disease after tuberculosis and influenza, and imposes a heavy burden on families and society. To inform future immunization policy, we investigated disease burden of varicella in China and explored cost-effectiveness of different varicella vaccination strategies at national and provincial levels.

**Methods:**

A dynamic transmission model was developed to assess disease burden of varicella and the impact of varicella vaccination in China. A cost-effectiveness analysis of three alternative vaccination strategies in China's National Immunization Program (NIP) compared with no vaccination was conducted. Scenario analyses and sensitivity analyses were performed to check the robustness of the results.

**Findings:**

It was estimated that 3.35 million new varicella cases occurred in 2019, more than three times of 982 thousand cases officially reported from National Notifiable Infectious Disease Surveillance System (NNIDSS). The under-reported rate was approximately 71%. The economic analysis revealed that from the societal perspective, the incremental cost-effectiveness ratio (ICER) for one dose of varicella vaccination in NIP was US$ 2357 per QALY at the national level and it was cost-effective in 22 of 31 provinces. The ICER for one dose varicella vaccination plus a mass catch-up for unvaccinated children aged 2–11 years old would be US$ −5260 per QALY, cost-saving at the national level. The one dose plus mass catch-up NIP strategy was also cost-saving in 24 of the 31 provinces.

**Interpretation:**

Varicella incident cases were substantially under-reported in China. Varicella vaccination in the NIP could significantly contribute to reducing the burden of varicella disease. From the societal perspective, including varicella vaccination into China's NIP was highly cost-effective at the national level and in most provinces.

**Funding:**

10.13039/100000865Bill & Melinda Gates Foundation.

**Research in context**

**Evidence before this study**

We searched PubMed in English and CNKI, Wanfang Data and CQVIP in Chinese for national and subnational varicella disease burden and economic analyses of varicella vaccines in China without date restrictions for all records matching “(chickenpox vaccine) or (varicella vaccine) or (varicella zoster virus) or (varicella) and (cost-effectiveness) or (cost-utility) or (cost-benefit) and (China)” in any field. No study of national and provincial estimates of varicella morbidity and cost-effectiveness analyses in English was identified in the literature. Seven Chinese studies were found to be related to cost-benefit analyses of varicella vaccines. Three of them used dynamic models and other four studies employed static models. However, no cost-effectiveness study was conducted for the entire country and at the subnational level. Several developed regions (e.g., Shanghai and Tianjin) had included varicella vaccines into their local immunization programs and had substantially increased vaccination coverage. Health disparities of varicella disease burden and vaccine coverage existed and future varicella vaccine policy urgently needed evidences from modelling of disease burden and economic analyses at the national and provincial levels.

**Added value of this study**

This is the first modelling and economic study in China to assess the varicella disease burden and cost-effectiveness of varicella vaccines at the national and provincial levels. More reliable estimates of vaccine-preventable varicella disease burden in China were modelled and compared with the official report from NNIDSS. Varicella cases of three alternative vaccination strategies in NIP were predicted from 2019 to 2049. National and provincial cost-effectiveness analyses were carried out for guiding future varicella vaccination policies in each of all 31 provinces in China.

**Implications of all the available evidence**

Including varicella vaccines into China's NIP could significantly reduce the disease burden of varicella. One dose varicella vaccine plus mass catch-up in NIP could be the most economical immunization strategy from the societal perspective. It would also be cost-effective to incorporate varicella vaccines into local immunization programs in most provinces in China.

## Introduction

Varicella zoster virus (VZV), a member of the Herpesviridae family, causes primary infection through inhalation of airborne saliva droplets or direct contact with the blistering fluid of a person with varicella or herpes zoster.^1^ The clinical manifestation of VZV infection is varicella, which is highly contagious with approximately 80%–90% of susceptible individuals being infected after exposure.^2^ As a self-limiting childhood disease, varicella typically causes mild symptoms such as skin lesions, fever, and loss of appetite.^3^ However, VZV infection may lead to serious consequences with complications including bacterial reinfection, pneumonia, cerebellar ataxia, and encephalitis.^4^ Despite the low incidence of these serious complications, they can still occur and result in hospitalization and even death.^5^ In 2014, the World Health Organization (WHO) estimated that there were approximately 140.0 million cases of varicella as the global disease burden each year, with 4.2 million serious complications leading to hospitalization and 4200 related deaths.^6^

Varicella vaccination is one of the most effective ways to prevent varicella. The varicella vaccine was firstly approved by the US Food and Drug Administration (FDA) in 1995 for use in children and adults aged 12 months with no history of varicella, and routine vaccination was initiated in 1996.^7^ After more than 30 years of clinical validation and scientific studies around the world, routine varicella vaccination in children had been proven to significantly reduce the morbidity, hospitalization, and mortality of varicella.^8^, ^9^, ^10^, ^11^ According to WHO, as of 2019, 50 out of the 194 WHO member states had included varicella vaccination into their childhood routine immunization programs.^12^

China had a high varicella disease burden. In 2019, varicella was the third most frequently reported vaccine-preventable infectious disease after tuberculosis and influenza according to National Notifiable Infectious Disease Surveillance System (NNIDSS). The real burden of varicella disease in China might severely be under-reported, as many people with varicella did not seek care in health facilities. Varicella could cause high economic and psychological burdens to individuals, families, and society.^13^^,^^14^ In China, varicella vaccination was firstly used in 1998.^15^ A systematic review found varicella vaccination coverage among children was about 61% in China in 2017.^16^ Varicella vaccination has not been incorporated into National Immunization Program (NIP) in China. Varicella vaccine is a voluntary self-paid vaccine available in the private market in China. Recently, a few provinces (e.g., Shanghai, Tianjin) and cities (e.g., Nanjing), included varicella vaccination into their local routine immunization programs for eligible children and achieved very high vaccination rates. To reduce the disease burden of varicella, and determine whether varicella vaccine should be included into routine immunization programs in China, it is necessary to investigate the real disease burden of varicella and economics of varicella vaccination at the national and subnational levels.

Cost-effectiveness analysis is commonly used to evaluate whether a vaccination strategy is economical. Economic evaluation of varicella vaccination was conducted in many countries such as the United States, Turkey, and the Netherlands.^17^, ^18^, ^19^ A dynamic transmission model of varicella was proposed by Halloran et al., in 1994 and had been used widely in the studies of varicella disease burdens.^20^ Epidemiological studies and economic evaluation of varicella were conducted using this model in France, Italy, and Germany.^21^, ^22^, ^23^ Brisson et al. optimized the model by incorporating the impact of the deterministic and realistic age structure on varicella transmission.^24^

However, there is no study focusing on varicella disease burden and cost-effectiveness analyses for varicella vaccination using a dynamic model in China, especially at the national and subnational levels. This study aims to fill this research gap. We investigate the disease burden of varicella and explore cost-effectiveness of three varicella vaccination NIP strategies at the national and provincial levels in China.

## Methods

### Disease burden model

#### Reported cases of varicella infection from National Notifiable Infectious Disease Surveillance System

A total of 40 notifiable infectious diseases are classified into three categories as A, B, and C according to China Infectious Disease Prevention and Control Law. National Notifiable Infectious Disease Surveillance System (NNIDSS) is a passive reporting system that obligates all health facilities in China to report all susceptible or confirmed cases of notifiable infectious diseases of above A, B, and C categories, when patients seek care in health facilities.^25^ NNIDSS is maintained by Chinese Center of Disease Control and Prevention at the national level. However, varicella does not belong to A, B, or C categories of notifiable infectious diseases in NNIDSS. Varicella is included into “other infectious diseases” voluntarily reported to NNIDSS.

Even if varicella was not officially included into notifiable infectious diseases in China and was only voluntarily reported, it was the third frequently reported vaccine-preventable infectious disease in NNIDSS after tuberculosis and influenza in 2019 with approximately one million reported cases. Because varicella was not an officially notifiable infectious disease in China, and data from NNIDSS might be severely under-reported.^26^ The present study collected more than 5.35 million reported cases of individual varicella from NNIDSS between 2010 and 2019. The national and provincial reported cases as well as their reported incidence rates in NNIDSS were analyzed to show dynamic trend of varicella disease burden in China. The reported cases of varicella were compared with estimated results of the dynamic transmission model, and showed under-estimated rates of real varicella disease burden in China.

### Dynamic transmission model

To assess real disease burden of varicella and the impact of varicella vaccination in China, a dynamic transmission model was developed in the Fortran programming language (Microsoft Visual Studio 2019). The results of transmission model were extracted into Microsoft Excel 2019 to conduct cost-effectiveness analyses.

The dynamic transmission model with realistic age structures and population changes was used to estimate the impact of vaccination on the disease burden of varicella. This model, originally developed by Halloran et al. (1994), estimated the impact of the varicella vaccination, modelling not only the number of varicella cases, but also the age distribution of cases.^20^ The present study employed this framework of the dynamic transmission model, and updated projections of the Chinese population were used. The model assumed no varicella related-death happened in China.^27^^,^^28^ Herpes zoster caused by varicella was also omitted in the model due to the fact that no data of herpes zoster were available in China.

**Fig. 1:**
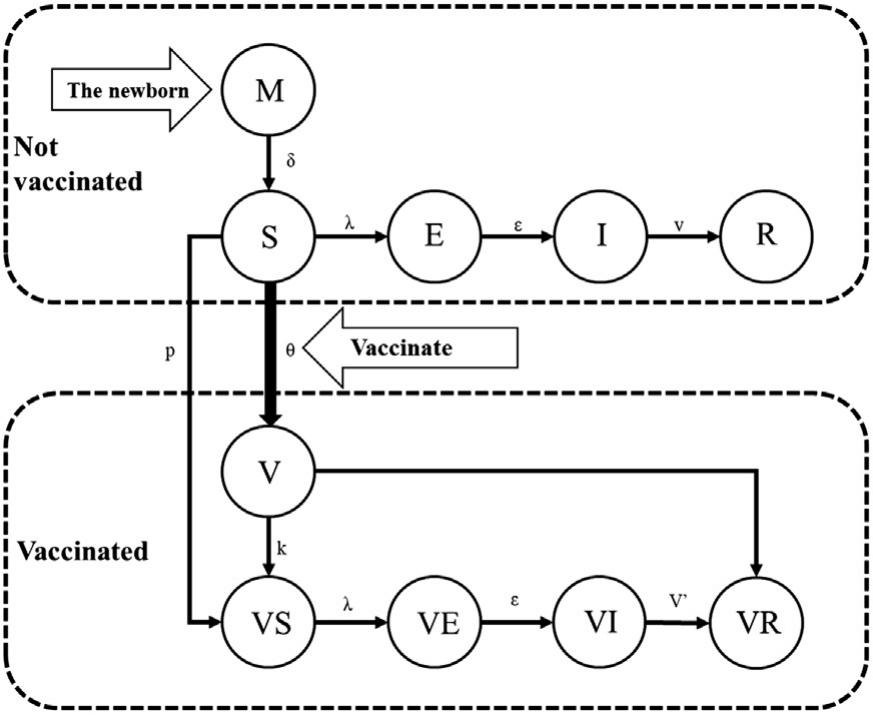
**Flow diagram of varicella transmission before and after vaccination.** Note: The mutually exclusive compartments represent different epidemiological states of varicella. The arrows represent the flow between these states. M = Maternal antibody; S = Susceptible situation; E = Exposed people who have latent or incubating infection; I = Infective situation; R = Recovery with high immunity; V = Vaccine-induced population immunity; VS = Susceptible situation after vaccination; VE = Latent situation after vaccination; VI = Infective situation after vaccination; VR = Recovery after vaccination.

The natural history of varicella disease was simulated based on the dynamic population structure in China, and the disease progression was divided into ten stages in the model. Each stage was described by a mathematical equation, and this led to a series of integral equations. The disease progression of varicella was illustrated in a simplified flowchart (Fig. 1), with each circle representing the possible states of an individual during the transmission of varicella. In this model, individuals were protected from infection at birth by maternal antibodies for an average of six months (state M), and then transferred to a healthy state of being "susceptible to varicella" (state S). If infection occurred, varicella had an average incubation period of 14 days (state E) and became infectious for seven days (state I) from the onset of varicella, after which the disease subsided (state R). With the introduction of varicella vaccination in the model, most children were protected after vaccination (state V). If vaccination failed or vaccine protection waned over time (into state VS), the vaccinated individuals might become infected (state VE and state VI), i.e., "breakthrough varicella cases". The average time from onset to recovery for breakthrough varicella cases was approximately 4.50 days.^24^^,^^29^

### Model inputs

Population data were obtained from the Sixth Population Census of China in 2010. In this study, the Leslie's model was used to simulate the population changes in China from 2019 to 2049, providing demographic data for the varicella transmission model.^30^ We also consider changes of family planning policies in China. The results of the provincial population census and population predictions were presented in Appendix Table S1.

With support from China CDC, the varicella vaccine coverage rates for children in ten provinces in 2019 were surveyed,^31^ and other provinces’ coverage rates were estimated according to the economic development of each province. These were used as varicella vaccine coverage rates for the private market in the model (Appendix Table S2). We used the data from domestic studies on effectiveness of varicella vaccines.^32^^,^^33^ The dynamic model in the present study also assumed that varicella vaccine effectiveness gradually decreased year by year, which had been supported by previous studies.^34^ Therefore, in the base case analysis, we used 90% and 92% as one dose and two doses effectiveness of varicella vaccines, respectively. In addition, more sensitivity analyses on effectiveness estimates of varicella vaccines i.e. 75% for one dose, and 90% for two doses, were taken according to meta-analysis articles in China.^35^^,^^36^ The density of exposure between age groups was derived from WAIFW (Who Acquires Infection From Whom) matrix by Brisson et al. (2000).^24^ The entire population in China was divided into five age groups considering their exposure density and infectious parameters: 0–4, 5–9, 10–14, 15–19, ≥20 years old. Natural transmission and disease course parameters of varicella were obtained from the previous literature.^29^ The specific parameters were listed in Appendix Table S3.

### Vaccination strategies

In this study, three alternative vaccination strategies in China's NIP were simulated. The first strategy was one dose varicella vaccine NIP strategy. Children would receive one dose of live attenuated varicella vaccine at one year old. The present study assumed that the coverage of the varicella vaccine would be 95% for NIP, as the high vaccination coverage was often achieved for all NIP vaccines in China.^37^ The second strategy was one dose varicella vaccine plus mass catch-up NIP strategy. This strategy involved a mass catch-up vaccination for unvaccinated children aged 2–11 years old in the first year of the NIP in 2019 besides the routine varicella vaccination for one-year-old children. The third was a strategy with two doses varicella NIP vaccination. This involved a second dose at the age of four in addition to a single dose for one-year-old children. The varicella vaccine analyzed in the present study was live attenuated varicella vaccine domestically made in China.

### Cost-effectiveness analysis

All above three NIP immunization strategies were evaluated from a societal perspective by comparing them with no varicella vaccination. Incremental cost-effectiveness ratios (ICERs) were calculated by costs and health effects per year. The ICERs showed the social cost of obtaining an additional quality-adjusted life years (QALYs), calculated as the difference in costs divided by the difference in QALYs. In the base case, the consequences of varicella were assessed over a 30-year period (2019–2049). The costs and health effects were both discounted at a rate of 3% per year.^38^ Referring to WHO criteria, we used one time GDP per capita of each province in China as the threshold for assessing whether one NIP immunization strategy was cost-effective.^39^

The economic burden of varicella disease included direct medical costs, direct non-medical costs, and indirect costs. Direct medical costs covered outpatient visits, registration, examinations, medicines, health care, and other medical expenses during hospitalization, as well as the cost of medicines purchased from pharmacies. Direct non-medical costs included transportation, food, accommodation, and nutrition. Indirect costs referred to the cost of absence from work for patients and caregivers. For adolescent patients aged between 15 and 18, indirect costs of their parents for accompanying medical treatments were estimated to be one day instead of the entire disease course (seven days). Above data were obtained from a domestic survey study, and 2020 China Statistics Yearbook.^40^^,^^41^ Provincial outpatient and inpatient costs were further adjusted for GDP per capita, average healthcare costs per time, and health costs per capita by province. These data were obtained from the 2020 China Health Statistics Yearbook.^42^ All costs were adjusted to 2019 year by consumer price index (CPI) for inflation. Costs were reported in US dollar (US$), and the average exchange rate in 2019 (1 US$ = 6.9 Chinese Yuan) was used.

The market prices of varicella vaccines from eight domestic manufacturers in 2019 were averaged with their market shares as weights to calculate the average cost of varicella vaccines. For other direct or indirect vaccination costs, we used data from China CDC, and examined the costs of each stage of the process from procurement to vaccination, such as administering costs and labor losses.^43^ The loss of QALYs due to natural and breakthrough cases of varicella was assumed to be uniform values nationwide. The loss of QALYs due to varicella equaled to the multiplication of the utility loss per day and the average number of sick days due to varicella. The utility loss due to varicella was measured by EuroQol-5 Dimension (EQ-5D) instrument.^40^ These specific parameters were shown in Table 1.

**Table 1:** Province-specific model parameters and data sources.

Model parameter	Age	Base case value	Source
**Population at risk and demographic parameters**			
Population at risk	Entire	Province-level data	National Bureau of Statistics,^41^ population prediction model^30^
Age-specific mortality rate (d)	0–4	9.05/1000	2020 China Health Statistics Yearbook^42^
	≥5	7.11/1000	2020 China Health Statistics Yearbook^42^
**Epidemiologic data**			
Mean duration of protection through maternal antibodies (1/δ) (days)		180.00	Halloran et al. (1996)^29^
Force of varicella infection in absence of vaccination (λ)	0-	0.032179	
	1-	0.064359	
	2-	0.096539	
	3-	0.128718	
	4-	0.160898	
	5–9	0.198778	
	10–14	0.191471	
	15–19	0.135138	
	≥20	0.102531	
Duration of the latent period (1/ε) (days)		14.00	
Duration of the infectious period (1/v) (days)		7.00	
Duration of the breakout case infectious period (1/v') (days)		4.50	
Probability of hospitalization for complications in natural cases		0.62%	Brisson et al. (2003)^44^
**Vaccine effectiveness & coverage**			
1-dose effectiveness		90.00%	Wu et al. (2013)^32^
2-dose effectiveness		92.00%	Xu et al. (2019)^33^
Percent of individuals for which vaccine fails completely (p)		4.00%	Burken et al. (1997)^45^
Rate of decay of vaccine antibodies (1/k) (days)		3650	Burken et al. (1997)^45^
Varicella vaccine coverage in private market (θ)	1–6 years	Province-level data	China CDC^31^
Varicella vaccine coverage in national immunization program (θ)	1–11 years	95.00%	Assumed by varicella vaccine coverage rate in Shanghai
**Cost of illness (US$)**			
Cost per inpatient case		Province-level data	Pan et al. (2019)^40^ and 2020 China Health Statistics Yearbook^42^
Cost per outpatient case		Province-level data	
Cost per breakthrough case		Province-level data	
**QALY**			
QALY of natural case		0.996	Pan et al. (2019)^40^
QALY of breakthrough case		0.999	
**Immunization costs (US$)**			
Vaccine price per dose		20.00	Yu et al. (2018)^43^
Cost of immunization delivery per dose		8.34	Yu et al. (2018)^43^
Wastage rate		5.00%	Authors' assumption

### Scenario analyses and sensitivity analyses

Scenario analyses and sensitivity analyses were conducted to assess the impact of parameters on the study results. Scenario analyses included a NIP strategy of two doses plus mass catch-up, which included one dose varicella vaccination at one year old, mass catch-up varicella vaccination in 2019 for all the children aged 2–11 years old, and the second dose at the age of four. Sensitivity analyses were performed on both varicella vaccine prices and vaccination coverage, considering the possibility of a further decrease in vaccine prices after including varicella vaccines in NIP and the possibility of varying the target 95% vaccine coverage rate below and above. For COVID-19 pandemic, we also included a scenario analysis considering the possible decrease of varicella disease burden due to non-pharmaceutical COVID interventions in the future. In addition, we also explored the variability on discount rates and vaccine effectiveness by setting different discount rates (0%–8%) and using more conservative vaccine effectiveness estimates.

### Role of the funding source

The sponsor of this study had no role in the study design, data collection, data analysis, data interpretation, writing of the report, or the decision to submit for publication. All authors had full access to all data used in the study and the corresponding authors had final responsibility for the decision to submit for publication.

## Results

### Disease burden results

Table 2 showed the reported cases of varicella from NNIDSS in 31 provinces between 2010 and 2019. In 2010, 327,054 varicella cases were reported by NNIDSS, which increased to 981,699 in 2019. From 2010 to 2019, reported varicella cases were tripled. Even if varicella vaccination had been increasing in China during the same period, the reported cases from NNIDSS also increased. Therefore, it was highly possible that the reported cases of varicella by NNIDSS had been under-estimated. NNIDSS might only capture or report a small portion of varicella cases. In addition, some patients with varicella might not seek care in health facilities and recover by themselves. In 2019, Jiangsu, Guangdong, and Guangxi were the three top provinces that reported the most varicella cases in China. Surprisingly, Jiangsu Province had 147.56 thousand reported varicella cases and was the highest disease burden province reported in 2019. The reported varicella incidence rates (per 1000 population) at the national level also increased from 0.24 in 2010 to 0.70 in 2019. Even if varicella cases were under-reported by NIDDSS, they still showed an increasing trend of varicella disease burden in China at the national and provincial levels.

**Table 2:** Cases of varicella infection reported in NNIDSS in 2010–2019 and modelled in 2019 (thousand).

Province	2010	2011	2012	2013	2014	2015	2016	2017	2018	2019	2019^a^ (model)
Anhui	8.26	12.52	11.60	14.29	15.08	20.38	20.99	30.01	40.68	51.32	205.10
Beijing	18.34	20.10	16.86	14.39	13.10	12.49	12.03	13.87	13.71	13.76	14.43
Chongqing	4.28	6.13	6.48	8.84	11.68	18.27	19.02	26.24	37.04	41.64	88.31
Fujian	6.29	8.81	7.96	8.55	7.00	9.57	8.79	14.68	18.88	24.41	183.35
Gansu	8.36	8.13	7.10	9.47	8.83	9.61	9.32	12.74	14.08	17.64	164.82
Guangdong	25.55	41.29	29.42	39.58	42.43	56.41	58.97	118.00	108.32	117.11	143.67
Guangxi	5.80	8.47	8.96	12.69	15.33	20.71	29.52	45.53	46.41	55.84	134.72
Guizhou	12.15	11.75	12.21	15.41	17.82	20.11	18.80	26.04	23.00	24.04	112.03
Hainan	2.32	4.74	2.11	2.94	4.47	5.84	5.06	7.89	7.19	8.30	42.83
Hebei	14.43	15.19	10.67	10.28	11.04	13.65	14.80	19.75	22.88	28.81	159.23
Heilongjiang	9.12	8.51	6.42	5.62	5.21	5.60	5.61	6.78	7.83	9.60	48.29
Henan	13.21	17.60	11.67	12.98	14.24	18.68	19.96	24.23	31.06	35.21	129.00
Hubei	19.10	23.81	19.61	20.93	19.59	23.51	25.61	32.84	39.81	43.85	185.42
Hunan	13.87	19.04	18.44	19.45	21.11	24.07	23.93	33.01	39.48	41.91	191.94
Inner Mongolia	5.36	6.12	4.27	4.05	3.94	4.39	4.46	6.39	8.30	9.95	45.70
Jiangsu	12.04	15.10	15.27	17.48	18.87	23.56	31.60	68.17	128.42	147.56	172.94
Jiangxi	8.06	10.80	10.12	10.33	11.17	12.03	12.58	19.75	21.71	25.13	161.81
Jilin	7.60	7.93	6.75	5.12	5.09	5.17	5.45	7.50	8.55	9.16	45.97
Liaoning	15.07	16.05	11.49	10.98	10.47	11.57	11.74	15.46	17.02	18.46	21.78
Ningxia	2.81	4.22	3.90	4.24	4.61	4.29	4.58	4.98	5.67	7.29	33.42
Qinghai	1.21	1.90	1.18	1.47	1.75	2.20	1.81	3.02	3.14	4.34	28.62
Shaanxi	5.70	8.63	8.22	9.56	9.17	12.06	15.03	17.64	23.89	28.01	126.98
Shandong	17.75	20.88	15.07	18.02	16.05	17.97	17.57	22.29	25.92	30.55	227.12
Shanghai	17.65	19.61	15.63	16.04	16.53	17.34	18.10	23.36	21.18	17.75	17.14
Shanxi	4.70	6.51	5.70	5.85	5.75	6.41	6.03	8.08	8.62	11.28	72.98
Sichuan	23.33	24.30	20.39	24.04	19.76	23.34	24.45	29.08	35.61	42.33	152.76
Tianjin	8.34	7.80	6.49	6.80	6.68	6.36	6.34	7.87	9.77	10.89	11.24
Tibet	1.55	1.68	0.78	1.81	1.84	1.78	1.91	2.58	2.51	2.42	7.86
Xinjiang	9.98	11.10	9.03	10.63	10.18	11.76	10.93	18.55	21.86	21.20	135.69
Yunnan	8.12	10.98	11.42	14.15	13.36	15.79	17.96	23.12	30.08	34.32	154.00
Zhejiang	16.70	23.85	24.12	24.21	25.15	26.90	24.13	32.71	36.84	47.67	132.99
National	327.05	403.52	339.31	380.20	387.27	461.81	487.08	722.17	859.46	981.70	3352.14

a showed the model estimated varicella cases in 2019.

On the other hand, the dynamic model estimated that there were 3.35 million new varicella cases in China in 2019. Shandong had 227.12 thousand cases as the highest provincial varicella incidents in China in 2019, while Tibet had 7.86 thousand cases as the lowest provincial varicella incidents. Comparing varicella cases reported by NNIDSS and estimates by the dynamic transition modelling in 2019, Table 2 showed that only 29% of varicella cases were reported by NNIDSS and the under-estimated rate was approximately 71%. Therefore, the economic analyses of varicella vaccination in China could not use the reported varicella cases from NNIDSS as the disease burden, and the dynamic modelling approach of varicella disease burden was employed in the cost-effectiveness analyses.

**Fig. 2:**
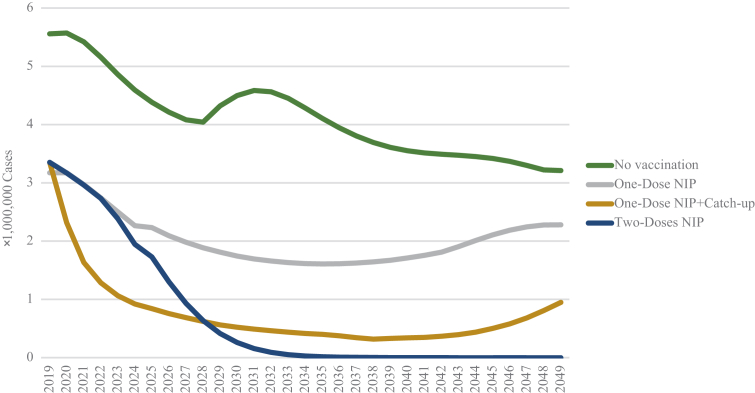
**Predicted varicella cases under four different immunization strategies in China, 2019–2049.**

Fig. 2 showed annual estimated cases of varicella from 2019 to 2049 for three different NIP vaccination strategies as well as no vaccination strategy. Since the total population in China was expected to decrease over the next 30 years and the proportion of child in the total population might decrease further, the total number of varicella cases would decrease year by year under all the vaccination strategies. The incident cases of the varicella were estimated to decrease more significantly in the first 20 years after varicella vaccine was introduced into NIP. The incidence rates were estimated to increase in the first ten years after vaccination, because there were still a huge number of unvaccinated population and the varicella vaccine was also not 100% effective with breakthrough cases. Small outbreaks often occurred when the proportion of the population without antibody protection reached a threshold. The outbreaks would steadily stabilize at a lower level of incidence after NIP vaccination. The incidence rates estimated by the dynamic model at the provincial level for three NIP vaccination strategies were shown in Appendix Table S3. One dose varicella vaccine in NIP strategy could prevent more than 61.74 million cumulative varicella cases over 30 years, compared with no vaccination. The reduced varicella cases by the one dose plus mass catch-up strategy and two doses vaccination strategy would be 98.85 million and 105.55 million in 30 years, respectively. Therefore, including varicella vaccines into China's NIP would substantially reduce varicella disease burden compared with no vaccination, and cost-effectiveness analyses of these NIP strategies were needed to show their economic features.

### Cost effectiveness results

As shown in Table 3, from the societal perspective, there would be 18,544 quality-adjusted life years (QALYs) loss per year in China from 2019 to 2049 without varicella vaccination at the national level. The QALYs loss would be decreased to 8303 if one dose varicella vaccination strategy was taken. The corresponding total costs per year were US$ 6312.80 million and US$ 6554.20 million respectively for above two strategies of no vaccination and one dose NIP. Compared with no varicella vaccination, one dose varicella vaccine in NIP with a coverage of 95% would be cost-effective at the national level. The ICER at the national level was US$ 2357 per QALY, which was much less than the one-time GDP per capita in 2019 in China (US$ 10,410). In addition, the one dose NIP strategy was cost-effective in 22 of 31 provinces, and their ICERs were much lower than one-time provincial GDP per capita in 2019.

**Table 3:** Cost-effectiveness analysis of three NIP vaccination strategies compared with no vaccination in 2019-2049

Province	No vaccination		One dose NIP			One dose plus mass catch-up NIP			Two doses NIP		
	Cost (US$ 1,000,000)	QALYs loss	Cost (US$ 1,000,000)	QALYs loss	ICER (US$ per QALY gained)	Cost (US$ 1,000,000)	QALYs loss	ICER (US$ per QALY gained)	Cost (US$ 1,000,000)	QALYs loss	ICER (US$ per QALY gained)
Anhui	236.35	902.76	299.72	470.78	14669.64	277.65	272.77	6556.42^a^	384.70	160.44	19985.10
Beijing	51.81	78.84	77.85	40.87	68606.22	79.30	29.01	55189.82	111.35	16.59	95645.39
Chongqing	152.93	452.01	133.72	165.37	Cost-saving^a^	115.94	77.74	Cost-saving^a^	178.41	77.74	6808.52^a^
Fujian	165.43	624.79	175.28	264.81	2737.51^a^	137.87	60.92	Cost-saving^a^	245.03	144.26	16565.46
Gansu	145.4	511.01	142.83	243.50	Cost-saving^a^	112.80	95.80	Cost-saving^a^	179.16	124.19	8725.12
Guangdong	281.51	904.91	427.1	419.57	29997.07	377.32	103.57	11956.35^a^	627.91	138.32	45186.29
Guangxi	362.57	1276.82	331.24	649.88	Cost-saving^a^	340.87	583.45	Cost-saving^a^	311.90	127.34	Cost-saving^a^
Guizhou	227.29	1014.65	228.87	527.95	325.75^a^	236.93	470.23	1771.54^a^	236.70	120.24	1052.62^a^
Hainan	47.58	209.27	52.67	111.45	5202.97^a^	50.49	80.32	2252.21	60.49	34.70	7396.36^a^
Hebei	267.75	896.85	324.86	426.66	12145.66	264.12	119.11	Cost-saving^a^	425.01	130.84	20530.17
Heilongjiang	135.54	313.94	144.38	112.95	4397.36^a^	119.14	17.56	Cost-saving^a^	222.21	74.63	36214.14
Henan	401.14	1307.39	457.78	617.38	8208.98	363.93	180.81	Cost-saving^a^	549.46	101.84	12303.20
Hubei	298.82	762.29	304.61	360.93	1442.07^a^	251.01	156.72	Cost-saving^a^	386.34	176.25	14933.22
Hunan	309.32	896.03	343.88	440.77	7590.95^a^	267.76	137.05	Cost-saving^a^	420.58	144.04	14795.42
Inner Mongolia	117.75	317.89	113.55	129.19	Cost-saving^a^	82.55	17.89	Cost-saving^a^	150.37	57.17	12512.35
Jiangsu	301.31	791.48	326.42	312.40	5240.64^a^	264.50	62.23	Cost-saving^a^	484.07	195.02	30639.25
Jiangxi	224.65	1011.05	244.93	498.71	3957.57^a^	218.35	278.57	Cost-saving^a^	287.11	128.84	7079.90^a^
Jilin	87.30	228.53	103.04	90.08	11370.00	86.32	16.20	Cost-saving^a^	161.21	64.79	45133.59
Liaoning	129.45	302.64	116.39	35.64	Cost-saving^a^	121.80	7.35	Cost-saving^a^	211.19	21.75	29102.28
Ningxia	46.87	138.46	43.36	71.15	Cost-saving^a^	34.50	35.57	Cost-saving^a^	45.99	26.33	Cost-saving^a^
Qinghai	39.89	113.75	36.28	55.92	Cost-saving^a^	26.76	21.28	Cost-saving^a^	40.08	22.34	213.56^a^
Shaanxi	200.45	576.90	182.35	235.31	Cost-saving^a^	132.4	46.89	Cost-saving^a^	242.78	130.58	9483.93^a^
Shandong	326.64	1023.33	382.65	397.89	8954.90^a^	319.98	74.31	Cost-saving^a^	568.61	202.47	29477.55
Shanghai	125.50	222.86	91.98	58.84	Cost-saving^a^	71.71	6.32	Cost-saving^a^	131.61	27.50	3128.61^a^
Shanxi	177.49	554.77	174.40	239.28	Cost-saving^a^	122.04	31.07	Cost-saving^a^	213.38	71.58	7428.71
Sichuan	259.06	761.94	300.04	259.67	8158.63	261.04	44.23	276.45^a^	464.42	134.31	32720.90
Tianjin	149.92	285.17	62.89	57.13	Cost-saving^a^	39.61	4.17	Cost-saving^a^	65.81	4.17	Cost-saving^a^
Tibet	4.23	46.32	10.17	20.79	23293.98	9.88	2.37	12862.85	17.02	6.38	32040.13
Xinjiang	161.73	533.43	159.50	291.49	Cost-saving^a^	146.59	206.62	Cost-saving^a^	166.11	98.53	1008.11^a^
Yunnan	238.50	966.45	254.40	480.61	3273.18^a^	207.94	205.99	Cost-saving^a^	294.01	132.61	6657.46^a^
Zhejiang	192.92	517.23	227.60	215.62	11498.97^a^	188.58	45.82	Cost-saving^a^	344.95	142.51	40571.57
National	6312.79	18543.75	6554.20	8302.57	2357.25^a^	5521.04	3491.95	Cost-saving^a^	8288.25	3038.31	12740.42

a Showed strategies were cost-effective, compared with a threshold of one-time GDP per capita in specific provinces.

The one dose plus mass catch-up NIP strategy would also save total social costs at the national level over the next 30 years, compared with no varicella vaccination. The ICER at the national level was US$ −5260 per QALY, so the one dose plus mass catch-up NIP strategy would be cost-saving at the national level. It would prevent 15,051.8 QALYs loss per year compared with no vaccination between 2019 and 2049. It could be cost-effective in 28 provinces as shown in Table 3, and only three provinces were not cost-effective. One dose plus mass catch-up NIP strategy was the most cost-effective among three NIP vaccination strategies studied in the present study.

The two doses varicella NIP strategy was the least cost-effective among three alternative vaccination strategies analyzed. The ICER of this strategy was US$ 12,740.42 per QALY. This strategy was not cost-effective at the national level, as it had the highest cost with US$ 8288.25 million per year. In addition, the two doses NIP strategy was cost-saving in only three provinces and cost-effective in nine provinces, so the provincial results were also less economical than the other two NIP strategies. However, Table 3 showed that the two doses NIP strategy was estimated to prevent the highest number of QALYs loss, as it could avoid about 15,505.44 QALYs annually between 2019 and 2049.

### Scenario analyses and sensitivity analyses results

The scenario analyses and sensitivity analyses results showed that the cost-effectiveness results at the national level were very robust and the provincial results varied across different influencing factors. Vaccine prices and effectiveness were two of most important factors for provincial results. The sensitivity analyses on vaccine prices showed that the results of cost-effectiveness at the national level were robust and more provinces would be cost-effective, even cost-saving, when the vaccine prices decreased from US$ 15 to US $5 per dose. Results on vaccine effectiveness at the national level were still robust and highly cost-effective, but more provinces would be not cost-effective if the first dose vaccine effectiveness decreased to 75%. Specific results of the scenario analyses and sensitivity analyses at the national level could be found in Table 4. Results of provincial sensitivity analyses were presented at Appendix Table S4–S9.

**Table 4:** Scenario analyses and sensitivity analyses on vaccine effectiveness compared with no vaccination.

		National	Number of provinces		
			Cost-saving	Cost-effective (ICER < one-time provincial GDP per capita)	Not cost-effective
Base case (Model-predicted incidence, one dose NIP, vaccination rate: 95%, and vaccine price: US$ 20.00 per dose)		Cost-effective	12	11	8
Strategy	Two doses	Not cost-effective	3	9	19
	One dose plus catch up	Cost-saving	25	5	1
	Two doses plus catch up	Not cost-effective	5	10	16
Coverage	99%	Not cost-effective	1	1	29
	90%	Cost-effective	12	13	6
	85%	Cost-effective	13	12	6
Vaccine price	US$ 15.00	Cost-saving	19	5	7
	US$ 10.00	Cost-saving	27	2	2
	US$ 5.00	Cost-saving	30	1	0
COVID-19 scenario	In the case of COVID-19	Not cost-effective	2	5	24
	55% reduction in incidence	Not cost-effective	2	0	29
	35% reduction in incidence	Not cost-effective	2	1	28
	5% reduction in incidence	Cost-effective	9	11	11
Discount rate	0%	Cost-effective	12	4	15
	5%	Cost-effective	12	12	7
	8%	Cost-effective	12	18	1
Vaccine effectiveness	One dose (75%)	Cost-effective	7	9	15
	First dose (75%) + Second dose (90%)	Not cost-effective	3	5	23

## Discussion

This is the first study to examine national and subnational disease burden of varicella and conduct cost-effectiveness analyses of varicella vaccines in China by using the dynamic model. First, the dynamic model showed very high varicella disease burden in China, which were much larger than those reported by NIDDSS. Second, we found significant reduction of varicella cases with all three NIP vaccination strategies, compared with no vaccination. Third, varicella vaccination was cost-effective for NIP strategies of one dose vaccination and one dose plus mass catch-up vaccination, compared with no varicella vaccination. However, the strategy of two doses NIP strategy was not cost-effective at the current price of varicella vaccines. Finally, for most provinces with high varicella disease burden, we found that including varicella vaccines into local immunization programs could be cost-effective, and even cost-saving.

One dose plus mass catch-up NIP strategy at the national level could prevent more than 61.74 million varicella cases over a 30-year period compared with no vaccination. Studies from other countries on the impact of varicella vaccination had similar results. In studies from United States, Netherlands, Spain, and other countries,^23^^,^^46^, ^47^, ^48^ dynamic models were used to study the changes of disease burden due to one dose varicella vaccination with a coverage of 90% or higher. One study from the United States found that both the one dose strategy and two doses strategy were be cost-saving from the societal perspective, compared with no vaccination.^47^ Another study from Spain also reported the routine varicella vaccination program for children was cost-saving.^48^ Varicella vaccination with high coverage rates were significantly effective in reducing varicella disease burden. These findings from developed countries implicated potential benefits from routine varicella vaccination program with low costs. We did a careful literature search, but did not found any studies about varicella diseases burden from developing countries using dynamic models. The present study showed all three vaccination NIP strategies could largely reduce the disease burden of varicella in China. The results from China might provide scientific evidence and policy implications for including varicella vaccination into NIP for other developing countries.

The economic analyses at the national level revealed that the one dose plus mass catch-up NIP varicella vaccination was cost-saving compared with no vaccination. Among three strategies studies in the present study, the most cost-effective one was the one dose plus mass catch-up NIP strategy. However, the economics of three strategies differed by provinces as different provinces currently had different morbidity rates. This suggested that if varicella vaccination could not be introduced in the NIP at the national level, different strategies could be considered for different provinces. In addition, we also found that the one dose plus mass catch-up NIP strategy was cost-saving in most provinces in China. However, only a few provinces in China had included varicella vaccination into their provincial vaccination programs, and none of them implemented the mass catch-up for children aged 2–11 years. These provinces might not obtain the highest economic benefit of varicella vaccination in local immunization programs.

The one-way sensitivity analyses on vaccine prices indicated that the cost of vaccination was an important factor in the economic evaluation. All the varicella vaccines used in the present market were made in China. According to previous experiences of other NIP vaccines in China, the prices of varicella vaccines could be significantly decreased, after varicella vaccines were successfully included in the NIP. Sensitivity analyses of vaccine effectiveness also showed the results were robust. Even though we used more conservative effectiveness estimates of varicella vaccines, one dose NIP strategy was still cost-effective at the national level. For most provinces with high varicella disease burden, including one dose varicella vaccine into the local immunization program was still highly cost-effective. However, results became not cost-effective for a few provinces with low varicella disease burden. Therefore, provincial immunization policies should be made according to epidemiological situation in specific provinces. In addition, we also did sensitivity analyses by using 0%, 5% and 8% per year as discount rates. The sensitivity analyses of various discount rates shown that the one dose NIP strategy was still cost-effective at the national level and the results were very robust. The provincial results varied after we changed the discount rates. The detailed provincial results with various discount rates could be found in Appendix Table S9.

There are several limitations in this study. First, the population model was projected over a 30-year time horizon, but unpredictable future population policies in China might cause significant variations in future age structures and fertility rates. Detailed data on the Seventh Population Census in China was still not available, so the Sixth Population Census was used. Moreover, the hospitalization rates, course length of varicella diseases, and other parameters for varicella used in the dynamic model might also change with future advances in medical treatment. Second, impacts of varicella vaccination on the incidence of herpes zoster was not considered in this study, since no data on herpes zoster from China were available. Third, the actual situation could change during the implementation of varicella vaccination due to the different contexts in different provinces. For example, vaccination rates in a short period might not reach 95% in the national or local immunization programs due to vaccine hesitancy. Finally, critical selection, information, and confounding biases might also affect the study results, particularly at the provincial level, although sensitivity analyses still showed robust results at the national level and the varicella vaccination in China's NIP would be highly cost-effective.

In China, varicella vaccination in NIP can significantly reduce the disease burden of varicella, and the strategy of one dose plus mass catch-up in NIP is presently cost-saving from the societal perspective. Public health policy makers from different provinces in China should contemplate their vaccination strategies, when they plan to include varicella vaccination into local childhood immunization programs in the future. The disease burden of varicella and cost-effectiveness analyses of varicella vaccination in China may help other developing countries to make their own immunization policies.

## Contributors

HF (Huangyufei Feng) and HZ (Haijun Zhang) did the analyses for the disease burden and cost-effectiveness analysis and wrote the first draft of the manuscript. HF (Hai Fang) designed the entire project and oversaw the analysis and manuscript writing. DY and CM prepared data of provincial vaccine coverage and varicella disease burden from NNIDSS in China. HZ (Haonan Zhang) helped to revise the manuscript. All coauthors provided feedback during the design and interpretation of the project. HF (Hai Fang) supervised the entire project. All coauthors contributed to revisions of the manuscript. HF (Huangyufei Feng) and HZ (Haijun Zhang) contributed equally as the co-first authors. HF (Hai Fang) contributed as the senior author.

## Data sharing statement

Detailed model inputs and results are available from this article. Some model inputs are also available from the online open access database. Individuals wishing to obtain complete model inputs and codes should submit requests to Hai Fang (hfang@hsc.pku.edu.cn).

## Declaration of interests

HF reports grant from the Bill & Melinda Gates Foundation. All other authors declare no competing interests.

## Appendix A Supplementary data

Supplementary Material

Captions for Supplementary Material
