# Supplementary material for: National and provincial burden of varicella disease and cost-effectiveness of childhood varicella vaccination in China from 2019 to 2049: a modelling analysis
Source: Lancet Reg Health West Pac. 2022 Nov 11;32:100639. doi: 10.1016/j.lanwpc.2022.100639 (PMC9918754; doi:10.1016/j.lanwpc.2022.100639)
Supplement: Supplementary Material [file mmc1.docx]

**National and provincial burden of varicella disease and cost-effectiveness of childhood varicella vaccination in China from 2019 to 2049:**

**a modelling analysis**

**Supplementary materials**

**Authors:**

Huangyufei Feng^§^, MPH^1,2^, Haijun Zhang^§^, MS^1,2^, Chao Ma, PhD ^3^, Haonan Zhang, BA^1,2^, Dapeng Yin^*^, PhD^4^, Hai Fang^*^, PhD^2,5,6^

**Affiliations:**

1. School of Public Health, Peking University, Beijing 100191, China

2. China Center for Health Development Studies, Peking University, Beijing 100191,

China

3. National Immunization Program, Chinese Center for Disease Control and Prevention, Beijing, 102206, China

4. Hainan Center for Disease Control and Prevention, Hainan, 570203, China

5. Peking University Health Science Center-Chinese Center for Disease Control and

Prevention Joint Center for Vaccine Economics, Beijing 100191, China

6. Institute for Global Health and Development, Peking University, Beijing 100871, China

**Keywords:** Varicella vaccine, Disease burden, Cost-effectiveness, China

**§Co-first authors contribute equally.**

**Corresponding authors information:**

Dapeng Yin, Hainan Center for Disease Control and Prevention, Hainan, 570203, China, Email: yindapeng@hainan.gov.cn

Hai Fang, China Center for Health Development Studies, Peking University, Beijing 100191, China, Email: hfang@hsc.pku.edu.cn, Tel: +86-10-8280-5702

**Contents**

[Appendix table 1. Predicted population at the national and provincial levels in China from 2010 to 2049 (million) 3](#_Toc116490237)

[Appendix table 2. Provincial socio-economic indicators, varicella vaccine coverage, and economic disease burden in 2019 5](#_Toc116490238)

[Appendix table 3. Varicella disease burden under different vaccination strategies 7](#_Toc116490239)

[Appendix table 4. Sensitivity analyses of different vaccination strategies 12](#_Toc116490240)

[Appendix table 5. Sensitivity analyses of different vaccination coverage rates 14](#_Toc116490241)

[Appendix table 6. Sensitivity analyses of different vaccine prices 16](#_Toc116490242)

[Appendix table 7. Sensitivity analyses of different varicella incidences (in the case of COVID-19) 18](#_Toc116490243)

[Appendix table 8. Sensitivity analyses of different vaccine effectiveness estimates 20](#_Toc116490244)

[Appendix table 9. Sensitivity analyses of different discount rates 22](#_Toc116490245)

[Appendix table 10. Consolidated Health Economic Evaluation Reporting Standards 2022 (CHEERS 2022) checklist 24](#_Toc116490246)

| Appendix table 1. Predicted population at the national and provincial levels in China from 2010 to 2049 (million) | | | | | | | | | |
| --- | --- | --- | --- | --- | --- | --- | --- | --- | --- |
| **Province/Year** | **2010** | **2019** | **2020** | **2025** | **2030** | **2035** | **2040** | **2045** | **2049** |
| Anhui | 58.19 | 63.65 | 63.88 | 64.47 | 64.41 | 63.96 | 63.05 | 61.38 | 59.59 |
| Beijing | 12.71 | 19.92 | 19.93 | 19.84 | 19.49 | 18.85 | 17.91 | 16.75 | 15.74 |
| Chongqing | 28.18 | 29.82 | 29.81 | 29.56 | 28.99 | 28.28 | 27.29 | 26.03 | 24.90 |
| Fujian | 36.04 | 39.13 | 39.23 | 39.48 | 39.32 | 38.78 | 37.75 | 36.16 | 34.62 |
| Gansu | 25.03 | 27.18 | 27.25 | 27.37 | 27.20 | 26.87 | 26.31 | 25.44 | 24.51 |
| Guangdong | 102.09 | 109.18 | 109.55 | 110.68 | 110.89 | 110.06 | 107.69 | 103.71 | 99.58 |
| Guangxi | 45.08 | 48.89 | 49.17 | 50.30 | 51.09 | 51.52 | 51.54 | 51.21 | 50.71 |
| Guizhou | 33.94 | 37.33 | 37.50 | 38.24 | 38.76 | 39.08 | 39.18 | 39.02 | 38.69 |
| Hainan | 8.52 | 9.41 | 9.46 | 9.67 | 9.79 | 9.85 | 9.82 | 9.69 | 9.51 |
| Hebei | 70.25 | 74.11 | 74.24 | 74.28 | 73.60 | 72.23 | 70.03 | 67.02 | 64.37 |
| Heilongjiang | 37.48 | 39.01 | 38.93 | 38.22 | 37.04 | 35.42 | 33.35 | 30.91 | 28.73 |
| Henan | 92.00 | 97.22 | 97.48 | 98.17 | 97.92 | 96.94 | 95.08 | 92.14 | 89.27 |
| Hubei | 55.96 | 60.62 | 60.75 | 60.97 | 60.39 | 59.18 | 57.34 | 54.89 | 52.61 |
| Hunan | 64.22 | 69.71 | 69.90 | 70.30 | 70.01 | 69.24 | 67.80 | 65.65 | 63.64 |
| Inner Mongolia | 24.11 | 25.38 | 25.40 | 25.29 | 24.88 | 24.18 | 23.16 | 21.84 | 20.62 |
| Jiangsu | 76.93 | 81.43 | 81.39 | 80.53 | 78.78 | 76.35 | 73.06 | 68.82 | 65.02 |
| Jiangxi | 43.64 | 47.52 | 47.70 | 48.33 | 48.53 | 48.34 | 47.73 | 46.62 | 45.44 |
| Jilin | 26.87 | 28.02 | 27.96 | 27.45 | 26.60 | 25.43 | 23.95 | 22.19 | 20.63 |
| Liaoning | 42.86 | 42.96 | 42.78 | 41.59 | 39.89 | 37.66 | 34.91 | 31.76 | 29.11 |
| Ningxia | 6.16 | 6.78 | 6.81 | 6.92 | 6.96 | 6.95 | 6.87 | 6.71 | 6.53 |
| Qinghai | 5.50 | 6.01 | 6.03 | 6.08 | 6.08 | 6.03 | 5.93 | 5.76 | 5.58 |
| Shaanxi | 36.70 | 38.74 | 38.74 | 38.47 | 37.79 | 36.78 | 35.34 | 33.49 | 31.81 |
| Shandong | 93.70 | 99.19 | 99.18 | 98.42 | 96.65 | 93.99 | 90.34 | 85.61 | 81.46 |
| Shanghai | 22.54 | 23.72 | 23.68 | 23.29 | 22.62 | 21.67 | 20.39 | 18.92 | 17.66 |
| Shanxi | 34.99 | 36.71 | 36.77 | 36.80 | 36.43 | 35.67 | 34.48 | 32.86 | 31.30 |
| Sichuan | 67.89 | 74.66 | 75.01 | 76.48 | 77.52 | 78.16 | 78.37 | 78.04 | 77.37 |
| Tianjin | 12.71 | 13.01 | 12.99 | 12.76 | 12.40 | 11.88 | 11.21 | 10.42 | 9.74 |
| Tibet | 2.88 | 3.13 | 3.13 | 3.14 | 3.13 | 3.08 | 3.00 | 2.89 | 2.78 |
| Xinjiang | 21.36 | 24.08 | 24.24 | 24.87 | 25.27 | 25.53 | 25.58 | 25.37 | 25.03 |
| Yunnan | 44.95 | 48.60 | 48.73 | 49.16 | 49.18 | 48.82 | 48.01 | 46.68 | 45.30 |
| Zhejiang | 53.21 | 57.06 | 57.09 | 56.85 | 56.03 | 54.65 | 52.50 | 49.57 | 46.83 |
| **National** | **1286.69** | **1382.18** | **1384.71** | **1387.98** | **1377.64** | **1355.43** | **1318.97** | **1267.55** | **1218.68** |

| Appendix table 2. Provincial socio-economic indicators, varicella vaccine coverage, and economic disease burden in 2019 | | | | | | | | | |
| --- | --- | --- | --- | --- | --- | --- | --- | --- | --- |
| **Province** | **Varicella vaccine coverage in 2019** | **GDP (US$ 1,000,000,000)** | **GDP per capita（US$）** | **Per capita consumption expenditure (US$)** | **Per capita consumption expenditure on health care (US$)** | **Local government health budget expenditure (US$ 1,000,000,000)** | **Cost per inpatient care (US$)** | **Cost per outpatient care (US$)** | **Cost per breakthrough case (US$)** |
| Anhui | 73% | 5302.0 | 8356.5 | 2733.9 | 212.8 | 98.2 | 329.8 | 72.7 | 36.2 |
| Beijing | 55% | 5053.0 | 23460.0 | 6148.3 | 534.2 | 76.3 | 827.8 | 182.5 | 90.9 |
| Chongqing | 75% | 3372.3 | 10832.6 | 2967.7 | 275.1 | 4.8 | 426.2 | 93.9 | 46.8 |
| Fujian | 55% | 6056.4 | 15305.6 | 3616.3 | 215.3 | 66.8 | 333.5 | 73.5 | 36.6 |
| Gansu | 27% | 1245.5 | 4713.5 | 2268.4 | 231.3 | 46.6 | 358.4 | 79.0 | 39.4 |
| Guangdong | 61% | 15381.6 | 13453.1 | 4142.1 | 252.9 | 225.7 | 391.9 | 86.4 | 43.0 |
| Guangxi | 73% | 3033.9 | 6137.7 | 2345.5 | 230.9 | 80.8 | 357.7 | 78.8 | 39.3 |
| Guizhou | 73% | 2395.6 | 6633.3 | 2111.4 | 182.1 | 76.4 | 282.2 | 62.2 | 31.0 |
| Hainan | 55% | 758.4 | 8072.4 | 2793.6 | 184.9 | 24.3 | 286.4 | 63.1 | 31.5 |
| Hebei | 55% | 5014.9 | 6621.1 | 2569.6 | 242.7 | 99.3 | 376.1 | 82.9 | 41.3 |
| Heilongjiang | 57% | 1944.7 | 5169.0 | 2587.4 | 351.0 | 44.9 | 543.9 | 119.9 | 59.7 |
| Henan | 83% | 7751.3 | 8055.4 | 2333.1 | 249.4 | 141.0 | 386.5 | 85.2 | 42.5 |
| Hubei | 73% | 6546.9 | 11055.2 | 3081.0 | 318.7 | 86.0 | 493.8 | 108.8 | 54.2 |
| Hunan | 73% | 5678.9 | 8220.0 | 2925.6 | 280.7 | 94.5 | 434.9 | 95.9 | 47.8 |
| Inner Mongolia | 57% | 2458.9 | 9693.2 | 2963.3 | 301.1 | 46.0 | 466.6 | 102.9 | 51.3 |
| Jiangsu | 76% | 14233.1 | 17658.1 | 956.8 | 309.5 | 129.4 | 479.6 | 105.7 | 52.7 |
| Jiangxi | 64% | 3536.8 | 7594.9 | 2521.5 | 180.6 | 90.1 | 279.9 | 61.7 | 30.7 |
| Jilin | 57% | 15961.0 | 6210.7 | 2582.2 | 310.6 | 40.2 | 481.2 | 106.1 | 52.9 |
| Liaoning | 55% | 3558.5 | 8170.1 | 3171.8 | 347.7 | 52.1 | 538.8 | 118.8 | 59.2 |
| Ningxia | 27% | 535.5 | 7745.3 | 2613.8 | 275.2 | 15.2 | 426.4 | 94.0 | 46.8 |
| Qinghai | 27% | 423.7 | 6997.3 | 2506.4 | 285.1 | 21.2 | 441.7 | 97.4 | 48.5 |
| Shaanxi | 68% | 3684.7 | 9521.3 | 2495.0 | 282.5 | 66.6 | 437.7 | 96.5 | 48.1 |
| Shandong | 76% | 10152.5 | 10093.2 | 2918.2 | 259.5 | 130.3 | 402.1 | 88.6 | 44.2 |
| Shanghai | 95% | 5450.8 | 22468.4 | 6515.0 | 457.8 | 70.5 | 709.4 | 156.4 | 77.9 |
| Shanxi | 68% | 2432.4 | 6532.0 | 2266.1 | 260.1 | 52.4 | 403.0 | 88.8 | 44.3 |
| Sichuan | 75% | 6659.4 | 7967.7 | 2762.6 | 276.4 | 134.8 | 428.3 | 94.4 | 47.0 |
| Tianjin | 95% | 2014.9 | 12910.1 | 4550.6 | 427.4 | 28.3 | 662.3 | 146.0 | 72.7 |
| Tibet | 27% | 242.5 | 6986.0 | 1861.3 | 74.2 | 17.6 | 114.9 | 25.3 | 12.6 |
| Xinjiang | 27% | 1942.4 | 7754.3 | 2485.2 | 246.5 | 43.2 | 381.9 | 84.2 | 42.0 |
| Yunnan | 73% | 3317.7 | 6849.1 | 2254.3 | 200.6 | 86.9 | 310.9 | 68.5 | 34.1 |
| Zhejiang | 55% | 8907.4 | 15374.8 | 4575.1 | 303.2 | 105.1 | 469.8 | 103.6 | 51.6 |
| **National** | **68%** | **141552.2** | **10103.5** | **3937.6** | **271.8** | **2345.4** | **428.8** | **94.5** | **47.1** |

| Appendix table 3. Varicella disease burden under different vaccination strategies | | | | | | | | | | |  |
| --- | --- | --- | --- | --- | --- | --- | --- | --- | --- | --- | --- |
| **Province** | **Year** | **No vaccination** | | | **1 Dose NIP^#^** | | **1 Dose plus catch-up NIP** | | **2 Doses NIP** | |  |
|  |  |  |  |  |  |  |  |  |  |  |  |
|  |  | **Incidence (per 100,000)** | | **Total cases**  **(thousand)** | **Incidence (per 100,000)** | **Total cases (thousand)** | **Incidence (per 100,000)** | **Total cases (thousand)** | **Incidence (per 100,000)** | **Total cases (thousand)** |  |
| Anhui | 2019 | 553 | | 351.74 | 322 | 205.10 | 322 | 205.10 | 322 | 205.10 |  |
|  | Average | 317 | | 200.61 | 188 | 118.64 | 117 | 74.15 | 10 | 6.62 |  |
|  | 2049 | 314 | | 187.40 | 281 | 167.16 | 20 | 11.82 | <1 | 0.01 |  |
| Beijing | 2019 | 107 | | 21.36 | 72 | 14.43 | 72 | 14.43 | 72 | 14.43 |  |
|  | Average | 94 | | 17.52 | 54 | 9.95 | 42 | 7.76 | 3 | 0.47 |  |
|  | 2049 | 41 | | 6.51 | 24 | 3.80 | 75 | 11.76 | <1 | <0.01 |  |
| Chongqing | 2019 | 541 | | 161.31 | 296 | 88.31 | 296 | 88.31 | 296 | 88.31 |  |
|  | Average | 358 | | 100.45 | 145 | 40.60 | 66 | 18.39 | 10 | 2.85 |  |
|  | 2049 | 311 | | 77.33 | 196 | 48.75 | <1 | 0.05 | <1 | 0.05 |  |
| Fujian | 2019 | 699 | | 273.32 | 469 | 183.35 | 469 | 183.35 | 469 | 183.35 |  |
|  | Average | 364 | | 138.84 | 167 | 63.93 | 39 | 14.89 | 15 | 5.91 |  |
|  | 2049 | 223 | | 77.11 | 169 | 58.54 | 58 | 20.06 | <1 | <0.01 |  |
| Gansu | 2019 | 676 | | 183.76 | 606 | 164.82 | 606 | 164.82 | 606 | 164.82 |  |
|  | Average | 427 | | 113.56 | 219 | 58.14 | 90 | 23.96 | 20 | 5.32 |  |
|  | 2049 | 350 | | 85.80 | 328 | 80.28 | 413 | 101.28 | <1 | <0.01 |  |
| Guangdong | 2019 | 207 | | 226.21 | 132 | 143.67 | 132 | 143.67 | 132 | 143.67 |  |
|  | Average | 186 | | 201.09 | 95 | 102.72 | 25 | 26.83 | 4 | 4.63 |  |
|  | 2049 | 122 | | 121.68 | 89 | 88.84 | 101 | 100.92 | <1 | 0.02 |  |
| Guangxi | 2019 | 475 | | 232.26 | 276 | 134.72 | 276 | 134.72 | 276 | 134.72 |  |
|  | Average | 558 | | 283.74 | 326 | 165.90 | 147 | 74.76 | 9 | 4.35 |  |
|  | 2049 | 555 | | 281.43 | 405 | 205.24 | 104 | 52.89 | <1 | <0.01 |  |
| Guizhou | 2019 | 554 | | 206.75 | 300 | 112.03 | 300 | 112.03 | 300 | 112.03 |  |
|  | Average | 583 | | 225.48 | 350 | 135.23 | 181 | 70.09 | 9 | 3.61 |  |
|  | 2049 | 596 | | 230.66 | 475 | 183.88 | 110 | 42.56 | <1 | <0.01 |  |
| Hainan | 2019 | 657 | | 61.81 | 455 | 42.83 | 455 | 42.83 | 455 | 42.83 |  |
|  | Average | 479 | | 46.50 | 288 | 27.96 | 133 | 12.91 | 14 | 1.38 |  |
|  | 2049 | 469 | | 44.66 | 349 | 33.24 | 173 | 16.50 | <1 | <0.01 |  |
| Hebei | 2019 | 311 | | 230.53 | 215 | 159.23 | 215 | 159.23 | 215 | 159.23 |  |
|  | Average | 279 | | 199.30 | 148 | 105.36 | 44 | 31.09 | 7 | 5.14 |  |
|  | 2049 | 236 | | 151.69 | 197 | 126.53 | 3 | 1.82 | <1 | <0.01 |  |
| Heilongjiang | 2019 | 215 | | 83.85 | 124 | 48.29 | 124 | 48.29 | 124 | 48.29 |  |
|  | Average | 199 | | 69.76 | 75 | 26.41 | 12 | 4.19 | 4 | 1.56 |  |
|  | 2049 | 88 | | 25.34 | 11 | 3.17 | <1 | 0.04 | <1 | <0.01 |  |
| Henan | 2019 | 246 | | 239.60 | 133 | 129.00 | 133 | 129.00 | 133 | 129.00 |  |
|  | Average | 303 | | 290.53 | 165 | 157.72 | 49 | 46.92 | 4 | 4.16 |  |
|  | 2049 | 307 | | 274.19 | 205 | 182.92 | 13 | 11.26 | <1 | <0.01 |  |
| Hubei | 2019 | 569 | | 344.99 | 306 | 185.42 | 306 | 185.42 | 306 | 185.42 |  |
|  | Average | 290 | | 169.40 | 152 | 88.99 | 71 | 41.54 | 10 | 5.98 |  |
|  | 2049 | 191 | | 100.27 | 207 | 109.07 | 359 | 189.09 | <1 | <0.01 |  |
| Hunan | 2019 | 468 | | 326.49 | 275 | 191.94 | 275 | 191.94 | 275 | 191.94 |  |
|  | Average | 291 | | 199.12 | 162 | 110.94 | 53 | 36.47 | 9 | 6.19 |  |
|  | 2049 | 265 | | 168.75 | 248 | 157.84 | 337 | 214.25 | <1 | <0.01 |  |
| Inner Mongolia | 2019 | 281 | | 71.38 | 180 | 45.70 | 180 | 45.70 | 180 | 45.70 |  |
|  | Average | 296 | | 70.64 | 130 | 30.94 | 18 | 4.31 | 6 | 1.47 |  |
|  | 2049 | 235 | | 48.51 | 102 | 21.13 | 11 | 2.24 | <1 | 0.02 |  |
| Jiangsu | 2019 | 422 | | 343.68 | 212 | 172.94 | 212 | 172.94 | 212 | 172.94 |  |
|  | Average | 233 | | 175.89 | 100 | 75.24 | 20 | 15.39 | 7 | 5.58 |  |
|  | 2049 | 165 | | 107.51 | 51 | 33.25 | 15 | 9.73 | <1 | <0.01 |  |
| Jiangxi | 2019 | 544 | | 258.62 | 341 | 161.81 | 341 | 161.81 | 341 | 161.81 |  |
|  | Average | 471 | | 224.68 | 264 | 126.15 | 158 | 75.45 | 11 | 5.22 |  |
|  | 2049 | 497 | | 225.72 | 346 | 157.07 | 70 | 31.69 | <1 | <0.01 |  |
| Jilin | 2019 | 280 | | 78.58 | 164 | 45.97 | 164 | 45.97 | 164 | 45.97 |  |
|  | Average | 202 | | 50.79 | 83 | 21.02 | 15 | 3.86 | 6 | 1.48 |  |
|  | 2049 | 109 | | 22.43 | 8 | 1.67 | <1 | 0.06 | <1 | <0.01 |  |
| Liaoning | 2019 | 77 | | 32.86 | 51 | 21.78 | 51 | 21.78 | 51 | 21.78 |  |
|  | Average | 180 | | 67.25 | 22 | 8.30 | 5 | 1.72 | 2 | 0.70 |  |
|  | 2049 | 136 | | 39.55 | 9 | 2.49 | <1 | 0.02 | <1 | <0.01 |  |
| Ningxia | 2019 | 549 | | 37.22 | 493 | 33.42 | 493 | 33.42 | 493 | 33.42 |  |
|  | Average | 449 | | 30.77 | 253 | 17.30 | 91 | 6.23 | 16 | 1.08 |  |
|  | 2049 | 445 | | 29.07 | 373 | 24.37 | 19 | 1.24 | <1 | <0.01 |  |
| Qinghai | 2019 | 530 | | 31.83 | 476 | 28.62 | 476 | 28.62 | 476 | 28.62 |  |
|  | Average | 424 | | 25.28 | 227 | 13.51 | 91 | 5.44 | 15 | 0.92 |  |
|  | 2049 | 428 | | 23.90 | 347 | 19.35 | 519 | 29.00 | <1 | <0.01 |  |
| Shaanxi | 2019 | 584 | | 226.35 | 328 | 126.98 | 328 | 126.98 | 328 | 126.98 |  |
|  | Average | 352 | | 128.20 | 156 | 56.66 | 32 | 11.56 | 11 | 4.10 |  |
|  | 2049 | 242 | | 76.94 | 113 | 35.96 | 35 | 11.09 | <1 | 0.01 |  |
| Shandong | 2019 | 421 | | 417.51 | 229 | 227.12 | 229 | 227.12 | 229 | 227.12 |  |
|  | Average | 244 | | 227.41 | 105 | 97.35 | 20 | 18.37 | 8 | 7.33 |  |
|  | 2049 | 174 | | 142.00 | 116 | 94.76 | 22 | 17.94 | <1 | 0.05 |  |
| Shanghai | 2019 | 203 | | 48.24 | 72 | 17.14 | 72 | 17.14 | 72 | 17.14 |  |
|  | Average | 231 | | 49.52 | 66 | 14.19 | 7 | 1.55 | 3 | 0.55 |  |
|  | 2049 | 74 | | 13.04 | 36 | 6.37 | <1 | 0.06 | <1 | <0.01 |  |
| Shanxi | 2019 | 331 | | 121.61 | 199 | 72.98 | 199 | 72.98 | 199 | 72.98 |  |
|  | Average | 350 | | 123.28 | 167 | 58.92 | 22 | 7.73 | 7 | 2.35 |  |
|  | 2049 | 288 | | 90.17 | 237 | 74.21 | 49 | 15.42 | <1 | <0.01 |  |
| Sichuan | 2019 | 373 | | 278.42 | 205 | 152.76 | 205 | 152.76 | 205 | 152.76 |  |
|  | Average | 219 | | 169.32 | 82 | 63.18 | 14 | 10.71 | 6 | 4.93 |  |
|  | 2049 | 176 | | 135.93 | 78 | 60.69 | 3 | 2.61 | <1 | <0.01 |  |
| Tianjin | 2019 | 193 | | 25.06 | 86 | 11.24 | 86 | 11.24 | 86 | 11.24 |  |
|  | Average | 539 | | 63.37 | 119 | 14.03 | 9 | 1.00 | 3 | 0.36 |  |
|  | 2049 | 199 | | 19.36 | 265 | 25.80 | 1 | 0.11 | 1 | 0.10 |  |
| Tibet | 2019 | 280 | | 8.77 | 251 | 7.86 | 251 | 7.86 | 251 | 7.86 |  |
|  | Average | 338 | | 10.29 | 166 | 5.04 | 18 | 0.56 | 8 | 0.25 |  |
|  | 2049 | 319 | | 8.84 | 278 | 7.70 | 30 | 0.82 | <1 | <0.01 |  |
| Xinjiang | 2019 | 623 | | 149.98 | 563 | 135.69 | 563 | 135.69 | 563 | 135.69 |  |
|  | Average | 471 | | 118.54 | 284 | 71.54 | 112 | 28.30 | 17 | 4.38 |  |
|  | 2049 | 471 | | 117.75 | 379 | 94.82 | 32 | 7.98 | <1 | <0.01 |  |
| Yunnan | 2019 | 552 | | 268.21 | 317 | 154.00 | 317 | 154.00 | 317 | 154.00 |  |
|  | Average | 445 | | 214.77 | 252 | 121.53 | 151 | 72.57 | 10 | 4.97 |  |
|  | 2049 | 450 | | 203.79 | 348 | 157.60 | 92 | 41.76 | <1 | <0.01 |  |
| Zhejiang | 2019 | 376 | | 214.50 | 233 | 132.99 | 233 | 132.99 | 233 | 132.99 |  |
|  | Average | 213 | | 114.94 | 94 | 50.90 | 21 | 11.05 | 8 | 4.29 |  |
|  | 2049 | 158 | | 74.12 | 29 | 13.79 | 8 | 3.94 | <1 | 0.01 |  |
| **National** | **2019** | **402** | | **5556.79** | **243** | **3352.11** | **243** | **3352.11** | **243** | **3352.11** |  |
|  | **Average** | **308** | | **4120.83** | **154** | **2058.31** | **57** | **759.77** | **8** | **108.13** |  |
|  | **2049** | **264** | | **3211.44** | **187** | **2280.28** | **78** | **949.96** | **<1** | **0.34** |  |
|  |  |  |  |  |  |  |  |  |  |  |  |

| Appendix table 4. Sensitivity analyses of different vaccination strategies | | | | | | |
| --- | --- | --- | --- | --- | --- | --- |
|  | **2 doses NIP** | | **1 dose plus catch-up NIP** | | **2 doses plus catch-up NIP** | |
| **Province** | **QALYs loss** | **ICER*（US$ per QALY gained）** | **QALYs loss** | **ICER*（US$ per QALY gained）** | **QALYs loss** | **ICER*（US$ per QALY gained）** |
| Anhui | 160.44 | 19985.10 | 272.77 | **6556.42** | 50.65 | 17481.12 |
| Beijing | 16.59 | 95645.39 | 29.01 | 55189.82 | 4.38 | 80804.27 |
| Chongqing | 77.74 | **6808.52** | 77.74 | **cost-saving** | 23.40 | **4675.48** |
| Fujian | 144.26 | 16565.46 | 60.92 | **cost-saving** | 47.88 | **12450.36** |
| Gansu | 124.19 | 8725.12 | 95.80 | **cost-saving** | 42.61 | 4907.35 |
| Guangdong | 138.32 | 45186.29 | 103.57 | **11956.35** | 40.19 | 42394.57 |
| Guangxi | 127.34 | **cost-saving** | 583.45 | **cost-saving** | 32.91 | **cost-saving** |
| Guizhou | 120.24 | **1052.62** | 470.23 | **1771.54** | 27.10 | **628.94** |
| Hainan | 34.70 | **7396.36** | 80.32 | 2252.21 | 10.22 | **5944.40** |
| Hebei | 130.84 | 20530.17 | 119.11 | **cost-saving** | 41.58 | 19175.54 |
| Heilongjiang | 74.63 | 36214.14 | 17.56 | **cost-saving** | 16.75 | 26239.83 |
| Henan | 101.84 | 12303.20 | 180.81 | **cost-saving** | 30.75 | 13420.05 |
| Hubei | 176.25 | 14933.22 | 156.72 | **cost-saving** | 52.50 | **9269.01** |
| Hunan | 144.04 | 14795.42 | 137.05 | **cost-saving** | 46.81 | 12884.30 |
| Inner Mongolia | 57.17 | 12512.35 | 17.89 | **cost-saving** | 14.66 | **9218.64** |
| Jiangsu | 195.02 | 30639.25 | 62.23 | **cost-saving** | 51.74 | 22034.43 |
| Jiangxi | 128.84 | **7079.90** | 278.57 | **cost-saving** | 37.58 | **6607.52** |
| Jilin | 64.79 | 45133.59 | 16.20 | **cost-saving** | 15.54 | 31360.57 |
| Liaoning | 21.75 | 29102.28 | 7.35 | **cost-saving** | 7.11 | 31513.89 |
| Ningxia | 26.33 | **cost-saving** | 35.57 | **cost-saving** | 8.19 | **cost-saving** |
| Qinghai | 22.34 | **213.56** | 21.28 | **cost-saving** | 7.05 | **cost-saving** |
| Shaanxi | 130.58 | **9483.93** | 46.89 | **cost-saving** | 37.18 | **4924.47** |
| Shandong | 202.47 | 29477.55 | 74.31 | **cost-saving** | 60.92 | 24936.98 |
| Shanghai | 27.50 | **3128.61** | 6.32 | **cost-saving** | 5.89 | **1768.67** |
| Shanxi | 71.58 | 7428.71 | 31.07 | **cost-saving** | 20.69 | 6740.92 |
| Sichuan | 134.31 | 32720.90 | 44.23 | **276.45** | 40.93 | 29081.35 |
| Tianjin | 4.17 | **cost-saving** | 4.17 | **cost-saving** | 3.77 | **cost-saving** |
| Tibet | 6.38 | 32040.13 | 2.37 | 12862.85 | 1.89 | 31111.65 |
| Xinjiang | 98.53 | **1008.11** | 206.62 | **cost-saving** | 32.10 | **cost-saving** |
| Yunnan | 132.61 | **6657.46** | 205.99 | **cost-saving** | 37.70 | **5853.62** |
| Zhejiang | 142.51 | 40571.57 | 45.82 | **cost-saving** | 40.03 | 29093.74 |
| **National** | 3038.31 | 12740.42 | 3491.95 | **cost-saving** | 890.71 | 10566.31 |
| *Compared with no vaccination. | | | | | | |
| Note: Numbers in bold black were cost-effective, compared with a threshold of one-time GDP per capita in specific provinces. | | | | | | |

| Appendix table 5. Sensitivity analyses of different vaccination coverage rates | | | | | | | |
| --- | --- | --- | --- | --- | --- | --- | --- |
| **Province** | **99% coverage** | | **90% coverage** | | **85% coverage** | | |
|  | **QALYs loss** | **ICER*（US$ per QALY gained）** | **QALYs loss** | **ICER*（US$ per QALY gained）** | **QALYs loss** | **ICER*（US$ per QALY gained）** |  |
| Anhui | 452.88 | 54188.78 | 490.30 | 14450.23 | 508.99 | 14115.97 |  |
| Beijing | 39.68 | 69872.19 | 42.88 | 68846.12 | 44.20 | 66394.39 |  |
| Chongqing | 157.08 | 20345.89 | 175.86 | **cost-saving** | 186.97 | **cost-saving** |  |
| Fujian | 252.61 | 31780.88 | 282.50 | **2712.82** | 296.62 | **2353.60** |  |
| Gansu | 230.96 | 25552.17 | 255.61 | **cost-saving** | 272.25 | **cost-saving** |  |
| Guangdong | 401.59 | 90691.95 | 440.19 | 29435.18 | 459.54 | 28639.05 |  |
| Guangxi | 633.39 | 17539.65 | 675.98 | **cost-saving** | 692.12 | **cost-saving** |  |
| Guizhou | 509.39 | 21887.47 | 551.37 | **306.43** | 568.90 | **cost-saving** |  |
| Hainan | 107.75 | 32172.78 | 111.77 | **3858.48** | 118.93 | **4480.25** |  |
| Hebei | 408.24 | 53277.32 | 447.25 | 11843.92 | 467.24 | 11448.64 |  |
| Heilongjiang | 110.78 | 54446.29 | 115.71 | **2555.82** | 119.05 | **796.09** |  |
| Henan | 568.77 | 43550.51 | 641.50 | **7585.83** | 664.55 | **6844.22** |  |
| Hubei | 344.79 | 40798.85 | 379.86 | **1351.96** | 398.21 | **1174.60** |  |
| Hunan | 421.82 | 48125.45 | 460.85 | **7343.93** | 480.94 | **7061.19** |  |
| Inner Mongolia | 123.80 | 32764.83 | 135.76 | **cost-saving** | 143.04 | **cost-saving** |  |
| Jiangsu | 302.03 | 49501.34 | 325.63 | **4203.57** | 340.29 | **3230.63** |  |
| Jiangxi | 481.54 | 29481.67 | 518.46 | **3642.52** | 538.20 | **3296.35** |  |
| Jilin | 88.59 | 63549.17 | 92.06 | 9476.20 | 94.26 | 7599.07 |  |
| Liaoning | 34.66 | 35786.05 | 37.06 | **cost-saving** | 38.61 | **cost-saving** |  |
| Ningxia | 68.48 | 22328.89 | 72.74 | **cost-saving** | 75.27 | **cost-saving** |  |
| Qinghai | 53.65 | 21674.82 | 58.64 | **cost-saving** | 61.13 | **cost-saving** |  |
| Shaanxi | 226.64 | 24353.74 | 246.31 | **cost-saving** | 257.79 | **cost-saving** |  |
| Shandong | 378.12 | 49922.73 | 421.03 | **8377.39** | 445.14 | **7817.85** |  |
| Shanghai | 56.60 | **16796.01** | 61.95 | **cost-saving** | 65.43 | **cost-saving** |  |
| Shanxi | 227.64 | 29375.31 | 253.06 | **cost-saving** | 266.69 | **cost-saving** |  |
| Sichuan | 247.73 | 51192.27 | 275.37 | **7301.93** | 292.38 | **6501.28** |  |
| Tianjin | 52.96 | **cost-saving** | 63.07 | **cost-saving** | 70.06 | **cost-saving** |  |
| Tibet | 19.74 | 55531.03 | 22.14 | 23328.15 | 23.33 | 23132.18 |  |
| Xinjiang | 282.44 | 27366.14 | 301.81 | **cost-saving** | 301.11 | **cost-saving** |  |
| Yunnan | 463.72 | 30394.76 | 500.09 | **2993.20** | 518.71 | **2626.67** |  |
| Zhejiang | 209.95 | 61903.04 | 222.92 | **10135.27** | 230.78 | **8791.48** |  |
| **National** | 7958.04 | 38179.88 | 8679.71 | **1851.38** | 9040.78 | **1237.00** |  |
| * Compared with no vaccination. | | | | | | | |
| Note: Numbers in bold black were cost-effective, compared with a threshold of one-time GDP per capita in specific provinces. | | | | | | | |

| Appendix table 6. Sensitivity analyses of different vaccine prices | | | |
| --- | --- | --- | --- |
| **Province** | **1 dose NIP** | | |
|  | **US$ 15 per dose** | **US$ 10 per dose** | **US$ 5 per dose** |
|  | **ICER*（US$ per QALY gained）** | **ICER*（US$ per QALY gained）** | **ICER*（US$ per QALY gained）** |
| Anhui | **7596.92** | **641.46** | **cost-saving** |
| Beijing | 44994.02 | 21773.27 | **cost-saving** |
| Chongqing | **cost-saving** | **cost-saving** | **cost-saving** |
| Fujian | **cost-saving** | **cost-saving** | **cost-saving** |
| Gansu | **cost-saving** | **cost-saving** | **cost-saving** |
| Guangdong | 19234.89 | **8651.13** | **cost-saving** |
| Guangxi | **cost-saving** | **cost-saving** | **cost-saving** |
| Guizhou | **cost-saving** | **cost-saving** | **cost-saving** |
| Hainan | 409.53 | **cost-saving** | **cost-saving** |
| Hebei | **4817.65** | **cost-saving** | **cost-saving** |
| Heilongjiang | **cost-saving** | **cost-saving** | **cost-saving** |
| Henan | 1503.86 | **cost-saving** | **cost-saving** |
| Hubei | **cost-saving** | **cost-saving** | **cost-saving** |
| Hunan | **329.64** | **cost-saving** | **cost-saving** |
| Inner Mongolia | **cost-saving** | **cost-saving** | **cost-saving** |
| Jiangsu | **cost-saving** | **cost-saving** | **cost-saving** |
| Jiangxi | **cost-saving** | **cost-saving** | **cost-saving** |
| Jilin | 2580.11 | **cost-saving** | **cost-saving** |
| Liaoning | **cost-saving** | **cost-saving** | **cost-saving** |
| Ningxia | **cost-saving** | **cost-saving** | **cost-saving** |
| Qinghai | **cost-saving** | **cost-saving** | **cost-saving** |
| Shaanxi | **cost-saving** | **cost-saving** | **cost-saving** |
| Shandong | 1769.59 | **cost-saving** | **cost-saving** |
| Shanghai | **cost-saving** | **cost-saving** | **cost-saving** |
| Shanxi | **cost-saving** | **cost-saving** | **cost-saving** |
| Sichuan | **720.24** | **cost-saving** | **cost-saving** |
| Tianjin | **cost-saving** | **cost-saving** | **cost-saving** |
| Tibet | 17537.50 | 11876.44 | **6215.39** |
| Xinjiang | **cost-saving** | **cost-saving** | **cost-saving** |
| Yunnan | **cost-saving** | **cost-saving** | **cost-saving** |
| Zhejiang | **2872.71** | **cost-saving** | **cost-saving** |
| **National** | **cost-saving** | **cost-saving** | **cost-saving** |

*Compared with no vaccination.Note: Numbers in bold black were cost-effective, compared with a threshold of one-time GDP per capita in specific provinces.

| Appendix table 7. Sensitivity analyses of different varicella incidences (in the case of COVID-19) | | | | | | | | |
| --- | --- | --- | --- | --- | --- | --- | --- | --- |
|  | **In the case of COVID-19** | | **Reduced incidence by 55%** | | **Reduced incidence by 35%** | | **Reduced incidence by 5%** | |
| **Province** | **QALYs loss** | **ICER*（US$ per QALY gained）** | **QALYs loss** | **ICER*（US$ per QALY gained）** | **QALYs loss** | **ICER*（US$ per QALY gained）** | **QALYs loss** | **ICER*（US$ per QALY gained）** |
| Anhui | 315.19 | 34191.00 | 211.85 | 63002.15 | 306.01 | 35962.98 | 447.24 | 16750.94 |
| Beijing | 20.72 | 196980.65 | 18.39 | 229963.93 | 26.57 | 139693.88 | 38.83 | 75554.63 |
| Chongqing | 103.95 | **8914.66** | 74.42 | 25603.49 | 107.49 | **7530.28** | 157.10 | **cost-saving** |
| Fujian | 184.09 | **15294.53** | 119.16 | 37742.62 | 172.12 | 18159.34 | 251.57 | **4244.91** |
| Gansu | 175.78 | 9372.58 | 109.57 | 31830.10 | 158.27 | 13483.90 | 231.32 | **448.44** |
| Guangdong | 226.78 | 81152.66 | 188.81 | 103542.12 | 272.72 | 62398.03 | 398.59 | 33164.08 |
| Guangxi | 508.79 | **1078.47** | 292.45 | 21777.56 | 422.42 | 6799.33 | 617.39 | **cost-saving** |
| Guizhou | 395.49 | 7513.59 | 237.58 | 26555.13 | 343.17 | 11881.35 | 501.56 | **1455.24** |
| Hainan | 87.46 | 12552.08 | 50.15 | 37959.75 | 72.44 | 19634.28 | 105.87 | **6613.55** |
| Hebei | 214.87 | 52532.08 | 192.00 | 62222.80 | 277.33 | 34207.62 | 405.33 | 14302.09 |
| Heilongjiang | 60.96 | 44602.37 | 50.83 | 62013.19 | 73.41 | 29780.56 | 107.30 | 6878.43 |
| Henan | 401.05 | 28431.16 | 277.82 | 54029.51 | 401.30 | 28395.65 | 586.51 | 10182.12 |
| Hubei | 163.43 | 48966.97 | 162.42 | 49507.37 | 234.60 | 22617.70 | 342.88 | **3511.87** |
| Hunan | 277.68 | 31434.97 | 198.35 | 57212.29 | 286.50 | 29452.10 | 418.73 | 9727.75 |
| Inner Mongolia | 84.52 | 15821.12 | 58.14 | 39504.24 | 83.97 | 16158.35 | 122.73 | **cost-saving** |
| Jiangsu | 198.12 | 29812.02 | 140.58 | 57307.60 | 203.06 | 28179.23 | 296.78 | **7482.76** |
| Jiangxi | 355.93 | 14049.02 | 224.42 | 34704.24 | 324.16 | 17503.31 | 473.78 | **5281.59** |
| Jilin | 43.50 | 63996.43 | 40.54 | 71437.12 | 58.55 | 37833.13 | 85.58 | 13956.62 |
| Liaoning | 19.26 | 27245.31 | 16.04 | 41292.42 | 23.16 | 15454.92 | 33.85 | **cost-saving** |
| Ningxia | 39.80 | 16432.91 | 32.02 | 28377.40 | 46.25 | 9584.70 | 67.59 | **cost-saving** |
| Qinghai | 43.84 | **1439.34** | 25.16 | 27776.02 | 36.35 | 8751.14 | 53.12 | **cost-saving** |
| Shaanxi | 155.09 | 9578.57 | 105.89 | 29858.05 | 152.95 | 10189.57 | 223.55 | **cost-saving** |
| Shandong | 230.14 | 38238.28 | 179.05 | 58056.88 | 258.63 | 30587.24 | 377.99 | 11069.34 |
| Shanghai | 30.42 | **cost-saving** | 26.48 | **cost-saving** | 38.25 | **cost-saving** | 55.90 | **cost-saving** |
| Shanxi | 163.62 | 12949.34 | 107.67 | 35834.12 | 155.53 | 15239.78 | 227.31 | **606.95** |
| Sichuan | 193.19 | 22468.93 | 116.85 | 58990.03 | 168.78 | 30552.88 | 246.68 | 10347.54 |
| Tianjin | 25.87 | **cost-saving** | 25.71 | **cost-saving** | 37.13 | **cost-saving** | 54.27 | **cost-saving** |
| Tibet | 12.02 | 46773.50 | 9.36 | 62631.85 | 13.51 | 40624.65 | 19.75 | 24987.96 |
| Xinjiang | 121.32 | 38490.40 | 131.17 | 33419.86 | 189.47 | 14208.56 | 276.92 | **558.42** |
| Yunnan | 451.39 | **5008.26** | 216.27 | 36031.61 | 312.39 | 17705.22 | 456.58 | **4683.83** |
| Zhejiang | 140.65 | 37207.84 | 97.03 | 70447.89 | 140.15 | 37469.47 | 204.84 | **14037.44** |
| *Compared with no vaccination. | | | | | | | | |
| Note: In the case of COVID-19, the reduction in incidence rates across provinces was assumed to have the same proportion as those in National Notifiable Infectious Disease Surveillance System in 2020. Numbers in bold black were cost-effective, compared with a threshold of one-time GDP per capita in specific provinces. | | | | | | | | |
|  | | | | | | | | |

| Appendix table 8. Sensitivity analyses of different vaccine effectiveness estimates | | |
| --- | --- | --- |
| **Province** | **Vaccine effectiveness & strategy** | |
|  | **1st dose（75% vaccine effectiveness）** | **1st dose (75% vaccine effectiveness) + 2nd dose (90% vaccine effectiveness)** |
|  | **ICER*（US$ per QALY gained）** | **ICER*（US$ per QALY gained）** |
| Anhui | 151821.67 | 21479.47 |
| Beijing | 579150.64 | 100068.77 |
| Chongqing | **cost-saving** | **8139.55** |
| Fujian | **36253.54** | 17892.65 |
| Gansu | **11747.70** | 9326.58 |
| Guangdong | 248751.45 | 46523.19 |
| Guangxi | **6475.23** | **cost-saving** |
| Guizhou | **40999.22** | **1836.74** |
| Hainan | 74191.80 | 8271.31 |
| Hebei | 120495.86 | 21411.16 |
| Heilongjiang | 43384.97 | 38428.74 |
| Henan | 104241.19 | 12952.94 |
| Hubei | **49014.14** | 17415.15 |
| Hunan | 102487.07 | 16282.06 |
| Inner Mongolia | **2858.28** | 13685.27 |
| Jiangsu | **58005.44** | 33623.83 |
| Jiangxi | 60549.59 | 7805.91 |
| Jilin | 93056.60 | 47873.49 |
| Liaoning | **cost-saving** | 29477.93 |
| Ningxia | **cost-saving** | **cost-saving** |
| Qinghai | **cost-saving** | **796.85** |
| Shaanxi | **cost-saving** | 11256.82 |
| Shandong | 85128.71 | 31453.47 |
| Shanghai | **cost-saving** | **4667.53** |
| Shanxi | **18549.79** | 8203.78 |
| Sichuan | 74890.95 | 34470.12 |
| Tianjin | **cost-saving** | **cost-saving** |
| Tibet | 177409.35 | 32307.47 |
| Xinjiang | **31537.52** | **1524.86** |
| Yunnan | 59209.34 | 7558.88 |
| Zhejiang | **98623.07** | 43263.19 |
| **National** | **49045.73** | 14005.13 |
| *Compared with no vaccination. | | |
| Note: Numbers in bold black were cost-effective, compared with a threshold of one-time GDP per capita in specific provinces. | | |

| Appendix table 9. Sensitivity analyses of different discount rates | | | |
| --- | --- | --- | --- |
| **Province** | **Discount rates** | | |
|  | **0%** | **5%** | **8%** |
|  | **ICER*（US$ per QALY gained）** | **ICER*（US$ per QALY gained）** | **ICER*（US$ per QALY gained）** |
| Anhui | 31414.20 | 9276.70 | **4587.04** |
| Beijing | 146916.34 | 43384.80 | **21452.42** |
| Chongqing | **cost-saving** | **cost-saving** | **cost-saving** |
| Fujian | **5862.23** | **1731.13** | **855.99** |
| Gansu | **cost-saving** | **cost-saving** | **cost-saving** |
| Guangdong | 64237.03 | 18969.37 | **9379.76** |
| Guangxi | **cost-saving** | **cost-saving** | **cost-saving** |
| Guizhou | **697.57** | **205.99** | **101.86** |
| Hainan | 11141.87 | **3290.23** | **1626.91** |
| Hebei | 26009.25 | 7680.60 | **3797.82** |
| Heilongjiang | 9416.71 | **2780.78** | **1375.01** |
| Henan | 17579.08 | **5191.15** | **2566.86** |
| Hubei | **3088.12** | **911.93** | **450.92** |
| Hunan | 16255.59 | **4800.32** | **2373.61** |
| Inner Mongolia | **cost-saving** | **cost-saving** | **cost-saving** |
| Jiangsu | **11222.54** | **3314.05** | **1638.69** |
| Jiangxi | 8474.92 | **2502.67** | **1237.49** |
| Jilin | 24348.21 | 7190.09 | **3555.27** |
| Liaoning | **cost-saving** | **cost-saving** | **cost-saving** |
| Ningxia | **cost-saving** | **cost-saving** | **cost-saving** |
| Qinghai | **cost-saving** | **cost-saving** | **cost-saving** |
| Shaanxi | **cost-saving** | **cost-saving** | **cost-saving** |
| Shandong | 19176.41 | 5662.85 | **2800.10** |
| Shanghai | **cost-saving** | **cost-saving** | **cost-saving** |
| Shanxi | **cost-saving** | **cost-saving** | **cost-saving** |
| Sichuan | 17471.24 | **5159.31** | **2551.11** |
| Tianjin | **cost-saving** | **cost-saving** | **cost-saving** |
| Tibet | 49882.75 | 14730.51 | 7283.77 |
| Xinjiang | **cost-saving** | **cost-saving** | **cost-saving** |
| Yunnan | 7009.33 | **2069.88** | **1023.49** |
| Zhejiang | 24624.39 | **7271.65** | **3595.60** |
| **National** | **5047.92** | **1490.66** | **737.09** |

*Compared with no vaccination.

Note: Numbers in bold black were cost-effective, compared with a threshold of one-time GDP per capita in specific provinces.

# Appendix table 10. Consolidated Health Economic Evaluation Reporting Standards 2022 (CHEERS 2022) checklist

**Items to include when reporting economic evaluations of health interventions**

It may be accessed via the *Value in Health* or via the ISPOR

Consolidated Health Economic Evaluation Reporting Standards 2022 (CHEERS 2022) webpage:

<https://www.ispor.org/heor-resources/good-practices/article/consolidated-health-economic-evaluation-reporting-standards-2022-cheers-2022-statement-updated-reporting-guidance-for-health-economic-evaluations>

| **Topic** | **No.** | **Item** | **Location where item is reported** |
| --- | --- | --- | --- |
| **Title** |  |  |  |
|  | 1 | Identify the study as an economic evaluation and specify the interventions being compared. | Page 1 |
| **Abstract** |  |  |  |
|  | 2 | Provide a structured summary that highlights context, key methods, results, and alternative analyses. | Page 3 |
| **Introduction** |  |  |  |
| **Background and objectives** | 3 | Give the context for the study, the study question, and its practical relevance for decision making in policy or practice. | Page 5-6 |
| **Methods** |  |  |  |
| **Health economic analysis plan** | 4 | Indicate whether a health economic analysis plan was developed and where available. | Page 8-9 |
| **Study population** | 5 | Describe characteristics of the study population (such as age range, demographics, socioeconomic, or clinical characteristics). | Page 8 |
| **Setting and location** | 6 | Provide relevant contextual information that may influence findings. | Page 8 |
| **Comparators** | 7 | Describe the interventions or strategies being compared and why chosen. | Page 8 |
| **Perspective** | 8 | State the perspective(s) adopted by the study and why chosen. | Page 8 |
| **Time horizon** | 9 | State the time horizon for the study and why appropriate. | Page 8 |
| **Discount rate** | 10 | Report the discount rate(s) and reason chosen. | Page 8 |
| **Selection of outcomes** | 11 | Describe what outcomes were used as the measure(s) of benefit(s) and harm(s). | Page 8 |
| **Measurement of outcomes** | 12 | Describe how outcomes used to capture benefit(s) and harm(s) were measured. | Page 8-9 |
| **Valuation of outcomes** | 13 | Describe the population and methods used to measure and value outcomes. | Page 8-9 |
| **Measurement and valuation of resources and costs** | 14 | Describe how costs were valued. | Page 8-9 |
| **Currency, price date, and conversion** | 15 | Report the dates of the estimated resource quantities and unit costs, plus the currency and year of conversion. | Page 9 |
| **Rationale and description of model** | 16 | If modelling is used, describe in detail and why used. Report if the model is publicly available and where it can be accessed. | Page 6-9 |
| **Analytics and assumptions** | 17 | Describe any methods for analysing or statistically transforming data, any extrapolation methods, and approaches for validating any model used. | Page 7, 9 |
| **Characterising heterogeneity** | 18 | Describe any methods used for estimating how the results of the study vary for subgroups. | Page 8 |
| **Characterising distributional effects** | 19 | Describe how impacts are distributed across different individuals or adjustments made to reflect priority populations. | Page 8 |
| **Characterising uncertainty** | 20 | Describe methods to characterise any sources of uncertainty in the analysis. | Page 9 |
| **Approach to engagement with patients and others affected by the study** | 21 | Describe any approaches to engage patients or service recipients, the general public, communities, or stakeholders (such as clinicians or payers) in the design of the study. | No applicable |
| **Results** |  |  |  |
| **Study parameters** | 22 | Report all analytic inputs (such as values, ranges, references) including uncertainty or distributional assumptions. | Table 1 |
| **Summary of main results** | 23 | Report the mean values for the main categories of costs and outcomes of interest and summarise them in the most appropriate overall measure. | Page 9-11 |
| **Effect of uncertainty** | 24 | Describe how uncertainty about analytic judgments, inputs, or projections affect findings. Report the effect of choice of discount rate and time horizon, if applicable. | Page 11 |
| **Effect of engagement with patients and others affected by the study** | 25 | Report on any difference patient/service recipient, general public, community, or stakeholder involvement made to the approach or findings of the study | No applicable |
| **Discussion** |  |  |  |
| **Study findings, limitations, generalisability, and current knowledge** | 26 | Report key findings, limitations, ethical or equity considerations not captured, and how these could affect patients, policy, or practice. | Page 11-13 |
| **Other relevant information** |  |  |  |
| **Source of funding** | 27 | Describe how the study was funded and any role of the funder in the identification, design, conduct, and reporting of the analysis | Page 9 |
| **Conflicts of interest** | 28 | Report authors conflicts of interest according to journal or International Committee of Medical Journal Editors requirements. | Page 9 |

From: Husereau D, Drummond M, Augustovski F, et al. Consolidated Health Economic Evaluation Reporting Standards 2022 (CHEERS 2022) Explanation and

Elaboration: A report of the ISPOR CHEERS II Good Practices Task Force. Value Health. 2022;25(1):10-31.
