# Supplementary material for: National and provincial burden of varicella disease and cost-effectiveness of childhood varicella vaccination in China from 2019 to 2049: a modelling analysis
Source: Lancet Reg Health West Pac. 2022 Nov 11;32:100639. doi: 10.1016/j.lanwpc.2022.100639 (PMC9918754; doi:10.1016/j.lanwpc.2022.100639)
Supplement: Captions for Supplementary Material [file mmc2.docx]

Caption for supplementary material

**Contents**

[Appendix table 1. Predicted population at the national and provincial levels in China from 2010 to 2049 (million) 3](#_Toc116490237)

[Appendix table 2. Provincial socio-economic indicators, varicella vaccine coverage, and economic disease burden in 2019 5](#_Toc116490238)

[Appendix table 3. Varicella disease burden under different vaccination strategies 7](#_Toc116490239)

[Appendix table 4. Sensitivity analyses of different vaccination strategies 12](#_Toc116490240)

[Appendix table 5. Sensitivity analyses of different vaccination coverage rates 14](#_Toc116490241)

[Appendix table 6. Sensitivity analyses of different vaccine prices 16](#_Toc116490242)

[Appendix table 7. Sensitivity analyses of different varicella incidences (in the case of COVID-19) 18](#_Toc116490243)

[Appendix table 8. Sensitivity analyses of different vaccine effectiveness estimates 20](#_Toc116490244)

[Appendix table 9. Sensitivity analyses of different discount rates 22](#_Toc116490245)

[Appendix table 10. Consolidated Health Economic Evaluation Reporting Standards 2022 (CHEERS 2022) checklist 24](#_Toc116490246)
